# Influence of the Environment on the Distribution and Quality of *Gentiana dahurica* Fisch.

**DOI:** 10.3389/fpls.2021.706822

**Published:** 2021-09-27

**Authors:** Mingxu Zhang, Dong Jiang, Min Yang, Tian Ma, Fangyu Ding, Mengmeng Hao, Yuan Chen, Chunhong Zhang, Xiaobo Zhang, Minhui Li

**Affiliations:** ^1^Baotou Medical College, Inner Mongolia, Baotou, China; ^2^State Key Laboratory Breeding Base of Dao-di Herbs, National Resource Center for Chinese Materia Medical, China Academy of Chinese Medical Sciences, Beijing, China; ^3^State Key Laboratory of Resources and Environmental Information System, Institute of Geographic Sciences and Natural Resources Research, Chinese Academy of Sciences (CAS), Beijing, China; ^4^College of Resources and Environment, University of Chinese Academy of Sciences, Beijing, China; ^5^Inner Mongolia Medical University, Hohhot, China; ^6^Inner Mongolia Hospital of Traditional Chinese Medicine, Hohhot, China; ^7^Inner Mongolia Key Laboratory of Characteristic Geoherbs Resources Protection and Utilization, Baotou, China

**Keywords:** *Gentiana dahurica* Fisch., environment, species distribution model, boosted regression trees, medicinal plant

## Abstract

*Gentiana dahurica* Fisch. is a characteristic medicinal plant found in Inner Mongolia, China. To meet the increase in market demand and promote the development of medicinal plant science, we explored the influence of the environment on its distribution and the quantity of its active compounds (loganic acid and 6’-*O*-*β*-D-glucosylgentiopicroside) to find suitable cultivation areas for *G. dahurica*. Based on the geographical distribution of *G. dahurica* in Inner Mongolia and the ecological factors that affect its growth, identified from the literature and field visits, a boosted regression tree (BRT) was used to model ecologically suitable areas in the region. The relationship between the content of each of active compound in the plant and ecological factors was also established for Inner Mongolia using linear regression. The results showed that elevation and soil type had the most significant influence on the distribution of *G. dahurica*—their relative contribution was 30.188% and 28.947%, respectively. The factors that had the greatest impact on the distribution of high-quality *G. dahurica* were annual precipitation, annual mean temperature, and temperature seasonality. The results of BRT and linear regression modeling showed that suitable areas for high-quality *G. dahurica* included eastern Ordos, southern Baotou, Hohhot, southern Wulanchabu, southern Xilin Gol, and central Chifeng. However, there were no significant correlations between the contents of loganic acid and 6’-*O*-*β*-D-glucosylgentiopicroside and the ecological factors. This study explored the influence of the environment on the growth and quantity of active compounds in *G. dahurica* to provide guidance for coordinating the development of medicinal plant science.

## Introduction

The geographical distribution of species is an important feature of different regions and is affected by environmental and human disturbances. Climatic and soil conditions are the main environmental factors that determine the distribution of species (Manthey and Box, 2007; Zhong et al., 2021). Species distribution models (SDMs) have been used to simulate geographical distributions and explore the environmental niche and potential distributions of species based on their spatial location and environmental variables. A variety of models have been established using different algorithms, such as Bioclim, Domain, genetic algorithm for rule-set prediction (GARP), MaxEnt, and boosted regression tree (BRT) (Stockwell and Peters, 1999; Phillips et al., 2006; Elith et al., 2008). BRT is an ensemble method for fitting statistical models; it is an additive regression model in which individual terms are simple trees, fitted in a forward, stepwise fashion. Although BRT models are complex, they can be summarized in ways that give powerful ecological insights, and their predictive performance is superior to most traditional modeling methods (Elith et al., 2008). The BRT model has been widely used in species distribution prediction and has achieved good results. For example, Gu et al. (2019) used BRT modeling to study the relationship between the subtropical climate and the growth of Masson pine (Gu et al., 2019). The analysis of different models for predicting Egyptian medicinal plant distribution by Kaky et al. (2020) included BRT. Yilmaz et al. (2017) use BRT to determine the factors affecting the distribution of *Muscari latifolium*. The use of BRT models to understand the relationship between different environmental factors and species distribution is, therefore, a useful approach for developing management and conservation strategies for medicinal plant species.

**FIGURE 1:**
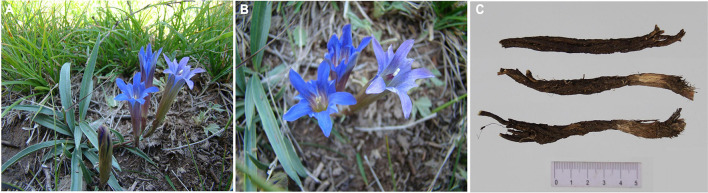
Wild *Gentiana dahurica* **(A)**; flowering *G. dahurica* **(B)**; dried roots of *G. dahurica* **(C)**.

*Gentiana dahurica* Fisch., known as xiaoqinjiao (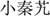) in China, is a perennial herb in the genus *Gentiana* sect. *Cruciata* (family Gentianaceae) (Figures 1A,B). Previous biogeographic studies have found that the Tibetan–Himalayan region of China has about 250 species (out of a total of about 400 species in the *Gentiana* genus) and is the main source area and center of diversity for *Gentiana* species. *Gentiana* species in the next most important biodiverse regions—Europe and North America—originated from the Himalayan region (Editorial Committee of Flora of China, 1993; Matuszak et al., 2016; Favre et al., 2016; Favre et al., 2020). Species of *Gentiana* are distributed throughout China, mostly in southwestern mountainous areas, alpine screes, alpine meadows, and scrubs. According to the Flora of China, *G. dahurica* is mainly distributed in northern Sichuan, northwestern China, northern China, and northeastern China, while a very similar species (*Gentiana campanulata* T. N. Ho) is only found in northwestern Sichuan. *Gentiana dahurica* grows on field margins, roadsides, sunny slopes, and steppes at an elevation of about 870–4,500 m (Editorial Committee of Flora of China, 1993). The dried root of *G. dahurica*, Gentianae Macrophyllae Radix, is a traditional Chinese medicine, which was first described and recorded in Shen Nong’s Herbal Classic (Han Dynasty, Shen Nong Ben Cao Jing), the earliest known monograph on herbal medicine in China, written some 2000 years ago (Figure 1C; Zhang et al., 2018). Gentianae Macrophyllae Radix is capable of removing dampness, cooling the body, relieving pain, and reducing fever; therefore, it is widely used for treating rheumatic diseases (Mirzaee et al., 2017; National Pharmacopoeia Committee, 2020). Modern medical research shows that gentidine is effective in treating formaldehyde-induced arthritis and inflammation caused by xylene according to the butyrylcholinesterase method. Gentiopicrin can increase the secretion of rat bile as well as promote gallbladder contraction, demonstrating a beneficial effect on the gallbladder. In addition, it has been reported that gencholine has an excitatory effect on the central nervous system of mice, while large doses of gencholine exhibit an anesthetic effect and can enhance the effects of barbiturate anesthesia.

Because of its broad range of applications, the unsustainable collection of wild *G. dahurica* increased rapidly. In 1987, *G. dahurica* was identified as a national wild protected plant by China, and artificial cultivation became the only legal source of *G. dahurica* (Huang et al., 2011; Nagahama, and Bonino, 2020). Although the government of Inner Mongolia has introduced policies to promote the development of medicinal plant cultivation, the scaling up of *G. dahurica* cultivation still faces many difficulties.^1^ An important reason for this is the unsuitable choice of planting areas (Cao et al., 2021). This research aims to (1) explore the influence of environment on the distribution of *G. dahurica* through an SDM and to identify suitable growing areas for *G. dahurica* in Inner Mongolia. This will provide a reference map for the conservation of wild *G. dahurica*; and (2) explore suitable growing areas for high-quality *G. dahurica* by establishing the relationship between active compounds and environmental factors, which can guide the development of the *G. dahurica* growing industry in Inner Mongolia.

## Materials and Methods

### Study Area Description

Inner Mongolia is located in the north of China between 97°10′18″–115°31′14″ E and 47°05′53″–37°24′26″ N, extending diagonally from the northeast to the southwest, in a long and narrow shape. It stretches 2,400 km from east to west, covering an area of 1.183 million km^2^. The region has a complex and diverse environment. Inner Mongolia has a continental monsoon climate, characterized by a significant decrease in precipitation and a substantial increase in evaporation from east to west, creating humid temperate, semi-humid, semi-arid, arid and extreme arid climatic zones. The regional landform presents a mosaic of plains, mountains, plateaus and basins (Zheng et al., 2019). The terrain changes from east to west, with many mountain ranges such as Yin Mountain and Helan Mountain. Many rivers such as Heilongjiang River, Liaohe River and the Yellow River flow through the territory (Huang, 2018). The Inner Mongolia region is inhabited by Mongolian, Han, Ewenki, Oroqen, Daur, Hui and Manchu ethnic minorities, composing the landscape of Mongolian traditional medicine which has unique applications for medicinal plants such as *G. dahurica* (Bi et al., 2019).

### Occurrence Records, Sample Collection, and Background Points

**FIGURE 2:**
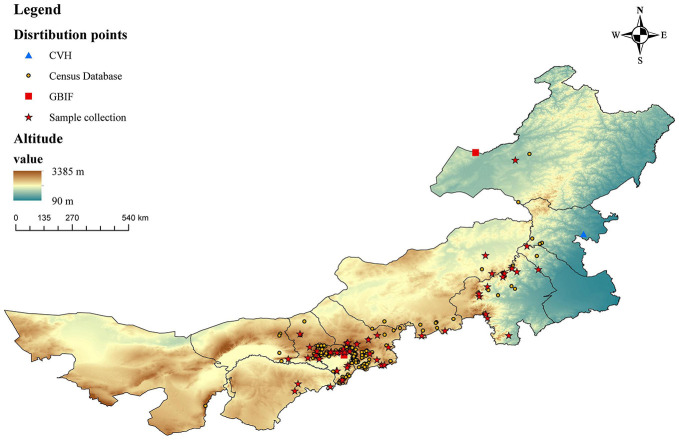
Geographical location of *Gentiana dahurica* sample points and the terrain in Inner Mongolia.

By searching the Chinese Virtual Herbarium,^2^ Global Biodiversity Information Facility,^3^ the Fourth National Survey of Chinese Medicine Resources and through field investigation, we collected a total of 291 distribution points for *G. dahurica* in Inner Mongolia (Figure 2). From these, 50 samples and related data were obtained through field investigation.

In order to sample the study area extensively, we first identified the biological characteristics and distribution of *G. dahurica* by consulting the Flora of China (Editorial Committee of Flora of China, 1993) and other monographs of medicinal plant databases of the China National Knowledge Infrastructure^4^ Wanfang^5^ Weipu^6^ and academic platforms such as Baidu Scholar^7^ and Google Scholar^8^. From these sources, a preliminary determination of the documented distribution area of *G. dahurica* was made. Then, using the village as the smallest survey unit, we checked with the local agricultural sector and farmers to determine where to collect *G. dahurica* samples. While collecting samples, handheld GPS units were used to record the latitude, longitude, and elevation of *G. dahurica*. Samples were collected from different areas with significant variations in environmental factors such as terrain, soil, and vegetation coverage in the survey area. The distance between each sample point was more than 1,000 m, and 20–30 strains of 3-year-old *G. dahurica* identified by experts were selected. Detailed sampling information is presented in Supplementary Table 1. For future experimental use, all samples were preserved in the herb depository of Inner Mongolia Key Laboratory of Characteristic Geoherbs Resources Protection and Utilization at Baotou Medical College.

### Selection of Ecological Factors

To reduce the influence of complex ecological factors on the construction of the model, we selected climatic factors, solar radiation data, topographic data and soil data with reference to botanical monographs and previous studies (Editorial Committee of Flora of China, 1993; Lu et al., 2016; Mirzaee et al., 2017; Guo et al., 2019). Moreover, in order to prevent the omission of ecological factors that may have a high impact on *G. dahurica* and to find the factors that have the greatest possible impact on the development of its cultivation industry, we did not eliminate these ecological factors (Table 1; Zheng et al., 2018). These were obtained from WorldClim^9^ version 2.1 climate data for 1,970--2,000 and the Resource and Environment Science and Data Center^10^ These environmental data were unified into a resolution of 30 s (∼1 km^2^) (Sillero and Barbosa, 2021).

**TABLE 1:** Details of the 15 ecological factors used to predict *G. dahurica* distribution.

**NO.**	**Name**	**Abbreviation**	**Type**	**Relative contribution**	**Ecological factor type**	**Data source**
1	Annual mean temperature	BIO1	Continuous	4.577%	Climatic factors	WorldClim, version 2.1
2	Mean diurnal range Mean monthly temperature (max temp—min temp)	BIO2	Continuous	0.539%		
3	Seasonality (standard deviation × 100)	BIO3	Continuous	10.091%		
4	Annual precipitation	BIO4	Continuous	14.567%		
5	Precipitation seasonality (coefficient of variation)	BIO5	Continuous	0.577%		
6	Solar radiation (mean)	Srad	Continuous	1.026%	Sun radiation	
7	Elevation	Elev	Continuous	30.188%	Terrain factor	
8	Vegetation types	Zblx	Categorical	5.071%		Resource and Environment Science and Data Center
9	Soil sand content	SOI1	Continuous	1.652%	Soil factor	
10	Soil moisture content	SOI2	Categorical	0.019%		
11	Soil clay content	SOI3	Continuous	0.004%		
12	Soil type	SOI4	Categorical	28.974%		
13	Cation exchange capacity	SOI5	Continuous	0.148%		
14	Soil organic carbon content	SOI6	Continuous	0.091%		
15	Annual sunshine duration	Suntime	Continuous	2.477%	Climatic factors	

### Boosted Regression Tree Model Analyses

#### Model Parameter Setting and Validation

The R version 3.3.3 statistical programming environment was used in combination with the extension packages (i.e., dismo, caret and gbm) to build the BRT model and evaluate simulation accuracy. The assembled contemporary occurrence records of *G. dahurica* were converted to grid units with a 1 × 1 km^2^ spatial resolution based on geographical coordinates. In this study, 246 grid units reflecting suitable environmental conditions were obtained. To map the environmental suitability of *G. dahurica*, the BRT modeling procedure also required background points as input data. Based on the literatures, areas where the annual precipitation was < 250 mm or > 400 mm, or where the elevation was < 800 m, were considered less suitable for planting *G. dahurica* and were used as the basis for screening the background points (Guo et al., 2019; Li et al., 2019; Low et al., 2020). A total of 246 background points reflecting unsuitable environmental conditions for growing *G. dahurica* were randomly selected. To reduce the influence of background points on the simulation, the step of randomly selecting background points was performed 200 times. During each iteration, 492 samples were constructed. An ensemble of 200 BRT models was fitted, and a 10−fold cross−validation method was used to avoid overfitting during the training process. The main parameters (i.e., tree.complexity = 4, learning.rate = 0.005, step.size = 10, and bag.fraction = 0.75) of the BRT models were tuned according to the experiences noted in previous studies (Jiang et al., 2019; Ding et al., 2020; Fan et al., 2020), and the other parameters were set at default values. To evaluate the accuracy and performance of the BRT models, threshold-independent receiver operating characteristic (ROC) analyses were used (Elith et al., 2006). In the present study, models with an area under the curve (AUC) index value of 0.75 or higher, were accepted as robust models (Shabnam et al., 2020). In addition, the relative contribution (RC) indicator was used to quantify the contribution of each spatial predictor variable to the BRT ensemble models; the RC can indicate the importance of environmental variables for the distribution of *G. dahurica*. The R codes for constructing the BRT models and for parameter adjustment shown above can be found on the website.^11^

#### Ecological Suitability of Sites for *G. dahurica* in Inner Mongolia

The ASC format model prediction output file obtained from BRT analysis was imported into ArcGIS software. The ASC was converted to raster files to display the results (Jenks, 1967; Jenks and Caspall, 1971). To guide cultivation of *G. dahurica* by the local government and farmers more effectively in the current environment and develop the medicinal effects based on the ecological suitability zoning results for *G. dahurica*, Inner Mongolia county-level administrative data were overlaid in ArcGIS.

#### Main Ecological Variables Affecting *G. dahurica*

In the model calculation results (section “Model Parameter Setting and Validation”), we simultaneously obtained the RC of each ecological factor for the distribution of *G. dahurica*. By analyzing the response curve of the ecological factors whose contribution rate and permutation importance were > 2%, we confirmed the influence of each ecological factor. The ecological factors with a total RC value greater than 95% were selected as the main ecological factors for further analyzing the distribution of high-quality *G. dahurica*.

### Determination of the Content of Index Components (Gentiopicroside and Loganic Acid) and Construction of the Relationship Between Them and the Main Ecological Factors

In accordance with the 2020 edition of the Chinese Pharmacopoeia (2020 edition) (National Pharmacopoeia Committee, 2020), we used high-performance liquid chromatography to determine the content of gentiopicroside, loganic acid, 6’-*O*-*β*-D-glucosylgentiopicroside, swertiamarin, gentiopicroside, and sweroside in all samples of *G. dahurica*. Gentiopicroside and loganic acid are the main active compounds in *G. dahurica* and important indicators of the quality of *G. dahurica* in the Chinese Pharmacopoeia (National Pharmacopoeia Committee, 2020). In addition, we used the stepwise regression to construct a model of the relationship between the active compounds and the main ecological factors, using the SPSS 25.0 statistical analysis software.

### Regional Suitability for High-Quality *G. dahurica* in Inner Mongolia

The ArcGIS grid calculator was used to calculate the relational equations established in section “Determination of the Content of Index Components (Gentiopicroside and Loganic Acid) and Construction of the Relationship Between Them and the Main Ecological Factors” and create the spatial layers of active compounds in *G. dahurica* in Inner Mongolia. Using the ArcGIS software, the layers obtained were overlaid on the ecological suitability distribution layer of *G. dahurica*, and the suitability distribution areas for the highest levels of active compounds in *G. dahurica*, were obtained. In order to provide scientific results that could be directly used by decision-making bodies for the development of the medicinal material industry, we used natural breaks to divide the final results into four categories. This is a commonly used clustering method in mapping that can minimize the differences within groups, maximize the differences between groups in the classification results, and better maintain the statistical characteristics of the data (Tang and Yang, 2012). Finally, to identify the distribution area suitable for the growth of high-quality *G. dahurica*, the administrative data of Inner Mongolia at the county level were overlaid on the spatial distribution layers of gentiopicroside and loganic acid in *G. dahurica*.

## Results

### Relative Contribution of the Spatial Predictor Variables

**FIGURE 3:**
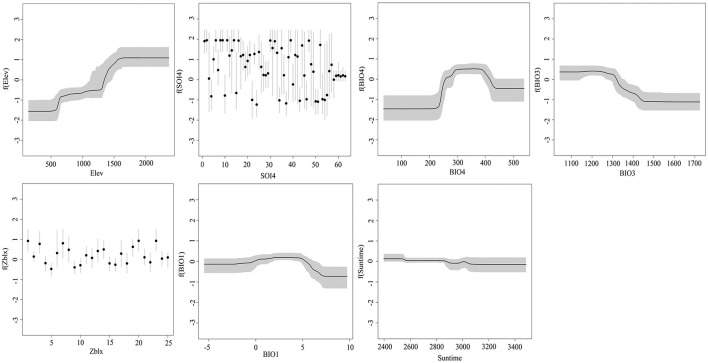
The marginal effect curves of the main spatial predictors (RC > 2.00%) over all 200 BRT ensemble models (The meaning of the numbering in SOI4 and Zblx is given in Supplementary Tables 3, 4).

Table 1 shows the RC of the related spatial predictor variables during the modeling analysis process. In this study, soil factors, accounting for 35.955% of the variation explained by the BRT ensemble models, were the most important predictor variables in the model, followed by climatic factors (RC 32.828%), all soil factors (RC 30.188%), and solar radiation factors (RC 1.026%). The most noteworthy predictor variables were, in decreasing order of RC values, Elev (RC 30.188%), SOI4 (RC 28.974%), BIO4 (RC 14.567%), BIO3 (RC 10.091%), Zblx (RC 5.071%), BIO1 (RC 4.577%), Suntime (2.477%). Figure 3 presents the marginal effect curves of the main spatial predictors (RC > 2.00%) over all 200 BRT ensemble models.

## Accuracy Evaluation of the Boosted Regression Tree model

The BRT model showed a good predictive performance (a 10-fold cross-validation of AUC = 0.962 ± 0.03). Moreover, the uncertainty of the spatial prediction measured using standard deviation values indicated that the uncertainty was generally low (Supplementary Figure 1).

### Regional Ecological Suitability for *G. dahurica* in Inner Mongolia

**FIGURE 4:**
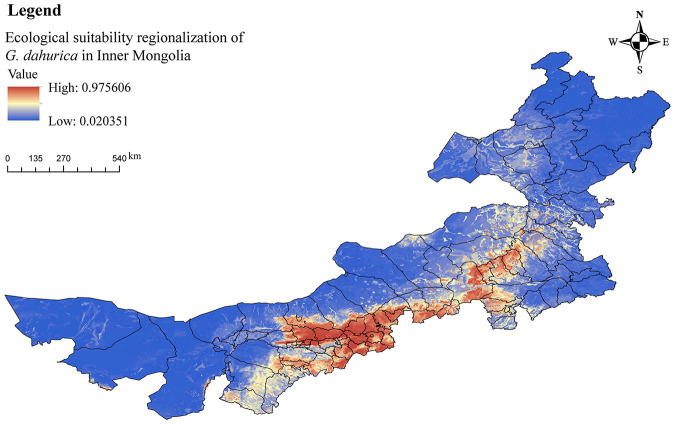
The regional ecological suitability for *Gentiana dahurica* in Inner Mongolia.

**FIGURE 5:**
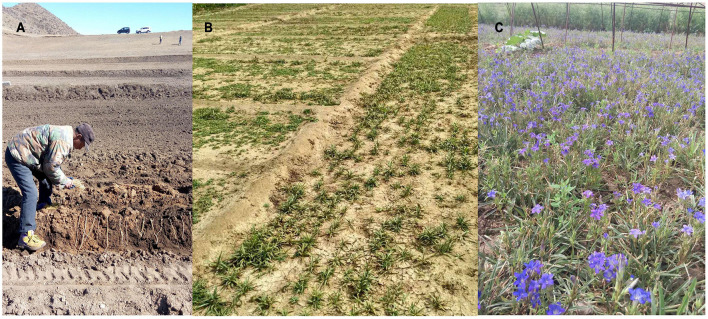
The production base of *Gentiana dahurica* in Wuchuan County. **(A)** The process of transplanting *G. dahurica* by farmers; **(B)** field of *G. dahurica*; **(C)** *G. dahurica* in bloom.

The experimental results showed that the most suitable areas for growing *G. dahurica* in Inner Mongolia were eastern Ordos, southern Baotou, Hohhot, southern Wulanchabu, southern Xilin Gol and central Chifeng (Figure 4). Field surveys in recent years showed that *G. dahurica* was planted in Wuchuan County of Hohhot and Siziwang Banner of Wulanchabu in Inner Mongolia, indicating that our experimental results are consistent with the actual production. Figure 5 shows the production base of *G. dahurica* in Wuchuan County.

### The Main Ecological Factors Affecting the Growth of *G. dahurica*

Seven main ecological factors among those screened (see section “Model Parameter Setting and Validation”) affected the growth of *G. dahurica*, with a total contribution of 95.945%. The response curves of *G. dahurica* to each of the main ecological factors show that elevation was conducive to the growth of *G. dahurica*. Soil type also had an important influence, although as shown in Figure 3, soil types had different effects on habitat suitability. The average annual precipitation had an important impact; the most suitable average annual precipitation was in the range of 250–400 mm. There was a negative correlation between suitable distribution of *G. dahurica* and temperature seasonality; the suitability remained relatively high below 1,300 but showed a sharp downward trend especially around 1,300–1,450.

### Active Compounds in *G. dahurica* and Their Correlation With the Main Ecological Factors

The contents of the active compounds index in the 50 samples of *G. dahurica* are shown in Supplementary Table 2. The contents of gentiopicroside and loganic acid met the limits set by the Chinese Pharmacopeia (2020 edition) (National Pharmacopoeia Committee, 2020). The equations showing the relationship between the gentiopicroside and loganic acid contents in *G. dahurica* and the main ecological factors were:

• *y*_1_ = 0.021 + 0.037x_1_ + 0.001x_2_ (*R*^2^ = 0.365, *P* ≤ 0.05; y_1_ = swertiamarin, x_1_ = BIO1, x_2_ = BIO4);
• *y*_2_ = −0.581 + 0.53 x_1_ + 0.021 x_2_ (*R*^2^ = 0.327, *P* ≤ 0.05; y_2_ = gentiopicroside, x_1_ = BIO1 x_2_ = BIO4);
• *y*_3_ = −0.05 + 0.000352 x_1_ (*R*^2^ = 0.349, *P* ≤ 0.05; y_3_ = sweroside, x_1_ = BIO4);
• *y*_4_ = 14.493 − 0.01 x_1_ + 0.02 x_2_ (*R*^2^ = 0.304, *P* ≤ 0.05; y_4_ = loganic acid and gentiopicroside, *x*_1_ = BIO3, x_2_ = BIO4);
• *y*_5_ = −1.208 + 0.697 x_1_ + 0.032 x_2_ (*R*^2^ = 0.336, *P* ≤ 0.05; y_5_ = total iridoids, x_1_ = BIO1, x_2_ = BIO4).

These equations suggest that the contents of all active compounds were positively correlated with the average annual precipitation (BIO4) to a certain extent, indicating that the increase in precipitation can promote the quantity of active compounds. Swertiamarin, gentiopicroside, and total iridoids have a positive correlation with the average annual temperature (BIO1), and an increase in temperature can effectively promote the quantity of these chemical compounds in *G. dahurica*. The amount of loganic acid and gentiopicroside was positively correlated with temperature seasonality (BIO3).

## Regional Suitability for High-Quality *G. dahurica* in Inner Mongolia

**FIGURE 6:**
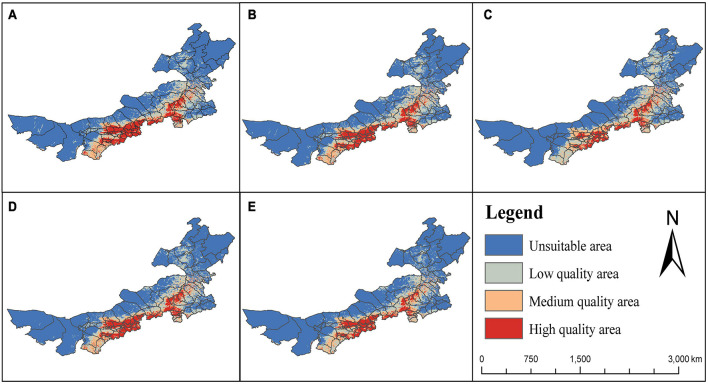
Regional suitability maps of high-quality *Gentiana dahurica* in Inner Mongolia. The suitability is based on swertiamarin **(A)**, gentiopicroside **(B)**, sweroside **(C)**, total amount of loganic acid and gentiopicroside **(D)**, and total iridoid **(E)** contents.

It can be seen from Figure 6 that the areas which are most suitable for high quantities of several compounds are similar. The overall trend shows an increase from northwest to southeast. The areas with high levels of chemical compounds are concentrated in the central and southern regions of Inner Mongolia. Therefore, cultivation of *G. dahurica* with high-quality active compounds is mainly suitable in eastern Ordos, southern Baotou, Hohhot, southern Wulanchabu, southern Xilin Gol, and central Chifeng, which are distributed around Yinshan and Daxinganling. These results are consistent with the BRT model prediction results indicating that elevation is an important factor affecting their distribution.

## Discussion

### Practical Application of Predictions

The present study shows that it is feasible to use SDMs to guide the introduction of medicinal plant cultivation, and the mapping can assist decision-making by government departments and farmers. For example, in a previous study, we examined the cultivation of *Astragalus membranaceus* var. *mongholicus* in Inner Mongolia, and the results showed that Baotou City was a suitable area. The results were consistent with the existing planting sites. Guyang County, Baotou City has been certified by the Ministry of Agriculture and Rural Affairs of the People’s Republic of China as a geographical indication product for agriculture, and the quality of *A. membranaceus* grown in this area has been recognized by the state and society.^12^ In a study over a larger area, Chen et al. (2020) used an SDM to predict the potential distribution of *Paeonia mairei*, which is an endemic plant to China, and provided guidance for the conservation of this species. Wang et al. (2021) studied the potential distribution of six Chinese almonds on a global scale to provide a reference for their wild species development and cultivation by exploring the relationship between environment and their distribution.

In the present study, the predicted suitable areas for cultivation and introduction of *G. dahurica* were eastern Ordos, southern Baotou, Hohhot, southern Wulanchabu, southern Xilin Gol, and central Chifeng. In agreement with field research conducted by the Alxa Comprehensive Experiment Station,^13^ we found that the current cultivation of *G. dahurica* in Inner Mongolia was mainly in Wuchuan County, Tumotzuo Banner, and Yuquan District, in the area around Hohhot, the administrative capital of Inner Mongolia (Supplementary Figure 2). This indicates that the predicted results of the study match the situation on-ground. Therefore, the other regions predicted in this study are also likely to be good locations for the development of a *G. dahurica* cultivation industry.

### Limitations of Sample Collection

According to the Flora of China (Editorial Committee of Flora of China, 1993), *G. dahurica* is distributed in various regions along the northeast to southwest of China, while a very similar species (*Gentiana campanulata* T. N. Ho) is only found in northwestern Sichuan. In this study, the samples were collected in Inner Mongolia and identified by botanical experts, which eliminated possible sample collection errors. Based on a long history of sample collection by Chinese researchers),^14^ we found that the specimen collection of *G. dahurica* was mainly distributed in the area from Shanxi to Qinghai. The specimens collected from Inner Mongolia were concentrated in the southern part of the region, which is consistent with the sampling of this present study. This suggests that our sampling is reliable. It is worth noting that there is some information on the distribution of separate existing sample points in the northeastern border area of Inner Mongolia, which indicates that the ecological environment of this area is also part of the distribution of *G. dahurica*. Sampling work for this area is worthy of attention in future studies, and may provide the boundary for planning the conservation of wild populations of *G. dahurica*.

Through the species distribution modeling approach, we were able to identify suitable and high-quality distribution areas for *G. dahurica* in Inner Mongolia, providing spatial guidance for conservation and industrial development of local populations. However, our study area was limited to the Inner Mongolia region, which is at the northern edge of the species’ range in China. This limits the generalization of some of the analysis and understanding of the species’ biology in this research. A survey of the literature found that the distribution of high-quality *G. dahurica* at the national scale remain unstudied. This may be related to the complex topography of northwestern and northern regions of China and the difficulty of collecting samples in the field on a large scale. The lack of knowledge about the growing area and environment of *G. dahurica* among different regions also poses a problem for larger scale sample collection. The present study offers a partial solution to this problem.

### Influence of Climatic Factors on the Distribution and Quality of *G. dahurica*

As shown in Figure 3, the climatic factors that had the strongest influence on the distribution of *G. dahurica* were BIO4 (14.567%) and BIO3 (10.091%). These factors, along with BIO1, were also important predictors of the medicinal quality of *G. dahurica*. This indicates that climatic factors, especially precipitation and temperature, predict locations that might be of particular interest to local farmers who wish to grow *G. dahurica*. This result is consistent with previous studies on the habitat suitability for *G. dahurica* using other models (Lu et al., 2016). It is noteworthy that soil type and elevation, which were important for the distribution of *G. dahurica*, were not significantly related to its active compound content. Therefore, when introducing this plant into cultivation in Inner Mongolia, a combination of yield and active compound content should be considered, and decisions should consider climatic factors, soil type, and elevation. The development of deep learning techniques and geospatial technology may provide more accurate models than multiple linear regression for the spatial simulation of variations in the active compounds of *G. dahurica* to express the relationship between environment and medicinal quality. The status of climate factors in Inner Mongolia is shown in Supplementary Figure 3.

### Effects of Soil Type and Elevation on the Distribution of *G. dahurica*

In most previous studies, only climatic factors were used as influencing factors to model species distribution; it is widely recognized that climate plays a major role in limiting species distributions at broad spatial scales. However, the homogeneity of climate factors at the landscape scale may affect the predicted distribution results, while soil type and elevation may show significant complexity at local scales which help model construction and interpretation of population prediction results (Figueiredo et al., 2018; Zuquim et al., 2020; Zhong et al., 2021).

The results of the study showed that the two ecological factors with the greatest influence on the distribution of *G. dahurica* suitability were elevation (30.188%) and soil type (28.974%). According to Figure 3 and Supplementary Figure 4, land above 1,500 m is the most suitable for *G. dahurica*. This range is similar to the results of the Flora of China and other studies, indicating that *G. dahurica* grows in higher elevation areas, such as mountain slopes and alpine meadows (Lu et al., 2016). According to Figure 3 and Supplementary Figure 5, Urban, Inland water, Calcaric Fluvisols, Umbric Gleysols, Calcic Kastanozems, Eutric Regosols, Eutric Cambisols, Gleyic Cambisols, Terric Histosols, Dystric Regosols, and Aric Anthrosols are suitable soil types for cultivating *G. dahurica*. The soil type of urban land showed a high suitability for *G. dahurica*, which is consistent with the soil type of Hohhot, the main planting area of *G. dahurica*, where the land type is mainly urban. *Gentiana dahurica* is also widely distributed on river banks, sandy areas around lakes, and along ditches. The other soil types are detailed soil subclasses, which can accurately show the influence of complex soil types on the distribution of *G. dahurica*. Although this very detailed information can lead to problems caused by sampling bias, all the above soil types are reference factors that farmers in Inner Mongolia should consider when starting to cultivate *G. dahurica*.

### Key Environmental Factors Affecting the Distribution of *G. dahurica* and the Formation of Secondary Metabolites

The medicinal material collected from plants cultivated in the highly suitable regions may not meet the minimum content requirement of active compounds. Researchers believe that the long-term adaptation of medicinal plants to specific stresses is an important condition for quality, and simulating the growth environment of wild medicinal plants is a way to guarantee the quality of medicinal herbs (Guo et al., 2020). However, the relationship between the accumulation of active compounds in *G. dahurica* and the environment has not yet been fully investigated. Studies have shown that the ecological environment suitable for cultivation of medicinal plants may not necessarily promote the accumulation of secondary metabolites in their tissues—these are the result of adverse conditions (Jiang et al., 2020). However, in this study, we found a high overlap between the suitable distribution areas of *G. dahurica* in Inner Mongolia and the areas where the active compounds were highest. It is possible that this is because *G. dahurica* in Inner Mongolia is at the edge of its overall distribution and, therefore, subject to environmental stressors from different climatic conditions. When referring to the climatic distribution pattern of the National Meteorological Science and Data Center species^15^ in China, it can be found that the ecological environment of Inner Mongolia is also at the edge of the ecological variation of the *G. dahurica* distribution area, which is quite different from that of its core distribution area. This may be why the cultivation of high-quality *G. dahurica* is expected from this province. The results of this study showed that although the suitable distribution area and high-quality distribution area of *G. dahurica* had many similarities, the ecological environmental factors affecting the two were different. Therefore, exploring the impact of the environment on the distribution of high-quality medicinal plants requires further research.

## Model Validity and Development of Multi-Species Distribution Models

In recent decades, a large number of ecological models have been developed for predicting potential suitable habitats for plants (Sun et al., 2020), based on the GIS techniques and statistical methods. Specifically, the predictions of ecological models can be used to evaluate suitable habitats for species and to provide a reference for detailed strategic planning to help solve the problems of ecological conservation and restoration (Elith et al., 2008). The distribution of medicinal plants and the accumulation of secondary metabolites are closely related to the environment. Sometimes, single SDMs are not able to predict the actual niche of medicinal plant species. The BRT model applied here uses R language, which is an open-source software, and provides clear guidance programs that researchers can use to better analyze scientific research results and help management agencies of the medicinal plant cultivation industry^16^. In this study, the AUC value obtained was 0.962 ± 0.03, indicating an accurate model. However, in further research, using machine learning models such as artificial neural networks or other methods may predict the distribution of different species more comprehensively.

## Conclusion

With the increasing demand for medicinal plants in China, the conservation of wild medicinal plant populations is under pressure, and the cultivation of medicinal plants has become a necessary measure. In this study, the BRT model indicated that the most important ecological factors driving the distribution of *G. dahurica* in Inner Mongolia were elevation, soil type, annual precipitation, seasonality, annual mean temperature, vegetation types, and annual sunshine duration. The relationships between active compounds and ecological factors in Inner Mongolia were modeled, and the most suitable areas for the development of a *G. dahurica* cultivation industry were predicted to be distributed around Yinshan and Daxinganling. This study provides a deeper understanding of the biogeographic distribution of *G. dahurica* and can be used to assist the government of Inner Mongolia Autonomous Region to conserve wild *G. dahurica* species and to develop a *G. dahurica* cultivation industry. Meanwhile, in future studies, it will be necessary to conduct a larger (documented biogeographic range or the whole China) and more comprehensive study on the sampling and suitable cultivation areas of *G. dahurica*, which to lay the foundation for a more deepened understanding of its role in the environment and for its sustainable use.

## Data Availability Statement

The original contributions presented in the study are included in the article/Supplementary Material, further inquiries can be directed to the corresponding author/s.

## Ethics Statement

Written informed consent was obtained from the individual (s) for the publication of any potentially identifiable images or data included in this article.

## Author Contributions

MZ and DJ conducted the experiment and analyzed the data. MY, TM, MH, YC, and CZ conducted the experiment. FD, XZ, and ML designed the experiment. All authors contributed to the article and approved the submitted version.

## Conflict of Interest

The authors declare that the research was conducted in the absence of any commercial or financial relationships that could be construed as a potential conflict of interest.

## Publisher’s Note

All claims expressed in this article are solely those of the authors and do not necessarily represent those of their affiliated organizations, or those of the publisher, the editors and the reviewers. Any product that may be evaluated in this article, or claim that may be made by its manufacturer, is not guaranteed or endorsed by the publisher.

## Footnotes

1
http://www.nmg.gov.cn/zwgk/zfxxgk/zfxxgkml/202012/t20201208315084.html

2
https://www.cvh.ac.cn/

3
https://www.gbif.org/

4
www.cnki.net

5
www.wanfangdata.com.cn

6
www.cqvip.com

7
xueshu.baidu.com/

8
scholar.google.com

9
https://www.worldclim.org/

10
http://www.resdc.cn/

11
https://rspatial.org/raster/sdm/9_sdm_brt.html

12
http://www.moa.gov.cn/nybgb/2017/dsq/201802/t20180201_6136210.htm

13
http://www.nmgzmyyy.com/yydt/info_9048.html

14
http://www.iplant.cn/info/Gentiana%20dahurica?t=s

15
http://data.cma.cn/data/weatherBk.html

16
https://rspatial.org/raster/sdm/9_sdm_brt.html
