# Supplementary material for: Influence of the Environment on the Distribution and Quality of Gentiana dahurica Fisch
Source: Front Plant Sci. 2021 Sep 27;12:706822. doi: 10.3389/fpls.2021.706822 (PMC8503573; doi:10.3389/fpls.2021.706822)
Supplement: Supplementary file 1 [file Table_1.DOCX]

Supplementary Material

# Supplementary Table S1

**Table S1.** Geographical distribution of 50 *G. dahurica* samples

| **NO.** | **League or City** | **Sample collection sites** | **Latitude** | **Longitude** |
| --- | --- | --- | --- | --- |
| S1 | Hulunbeir City | Huhenuoer of Bayanhada sumu in Chen Barag Banner, Hulun Buir | 119°11′22.16″ | 49°16′19.52″ |
| S2 | Tongliao City | Sanhe village of Dalaihushuo sumu in Holingola, Tongliao | 119°41′55.51″ | 45°34′06.24″ |
| S3 | Bayan Nur City | Dahuabei of Baiyanhua town in Urad Front Banner, Bayan Nur | 109°24′17.60″ | 40°42′3.71″ |
| S4 | Ordos City | Jiaqibu village of Hongqinghe town in Ejin Horo Banner, Ordos | 109°41′41.24″ | 39°19′27.61″ |
| S5 |  | Zhaizita of Kangbashen District in Ejin Horo Banner, Ordos | 109°49′46.98″ | 39°37′57.87″ |
| S6 |  | Miaojialiang village of Longkou town in Jungar Banner, Ordos | 111°13′7.49″ | 39°30′32.80″ |
| S7 | Xilingol League | Arihushu Gacha of Wulanhalaga sumu in West Ujimqin Banner, Xilingol League | 117°54′32.28" | 45°10′06.41" |
| S8 |  | Banla Mountain of Gurigesitai National Nature Reserve in Haoletugaole town in West Ujimqin Banner, Xilingol League | 118°11′40.74″ | 44°23′21.97″ |
| S9 |  | Baimaqun gacha of Gongbaolage sumu in Taibus Banner, Xilingol League | 116°09′51.62″ | 41°55′22.16″ |
| S10 |  | Houyingzi village of Dachengzi town in Ningcheng County, Chifeng | 115°09′57.20″ | 41°42′26.13″ |
| S11 | Chifeng City | Houyingzi village of Dachengzi town in Ningcheng County, Chifeng | 118°54′27" | 41°42′45" |
| S12 |  | Beidayingzi village of Dafuyingzi town in Songshan Disrict, Chifeng | 117°59′52.65" | 42°27′48.42" |
| S13 |  | Denglonghezi of Denglonghe Pasture in Ongniud Bannar, Chifeng | 117°55′12.36″ | 42°38′08.28″ |
| S14 |  | Dabaliang of Jingpeng town in Hexigten Banner, Chifeng | 117°38′35.22″ | 43°23′39.73″ |
| S15 |  | Dangzhongyingzi village of Yuzhoudi town in Hexigten Banner, Chifeng | 117°37′35.11″ | 43°31′11.56″ |
| S16 |  | Shangxigou village of Dayingzi town in Linxi County, Chifeng | 117°59′34.90″ | 43°49′08.99″ |
| S17 |  | Xiaomuhuangchuan of Hanshan Forest Farm in Balin Right Banner, Chifeng | 118°43′10.18″ | 44°23′13.87″ |
| S18 |  | Hanshangou village of Suoboriga town in Balin Right Banner, Chifeng | 118°38′56.38″ | 44°14′43.54″ |
| S19 |  | Fuhe village of Fuhe town in Balin left Banner, Chifeng | 119°15′32.08" | 44°28′03.47" |
| S20 |  | Milituba of Wulanba Nature Reserve in Balin Left Banner, Chifeng | 119°2′51" | 44°36′25" |
| S21 |  | Xinaili gacha of Saihantala sumu in Ar Horqin Banner, Chifeng | 120°10′48.36″ | 44°33′42.82″ |
| S22 | Baotou City | Tongyangdao street of Bayan Obo Mining District in Baotou | 109°54′33.83″ | 41°46′54.83″ |
| S23 |  | Maanshan of Xishihao town in Guyang County, Baotou | 110°36′1.40" | 40°52′39.65" |
| S24 |  | Gongzhongqu village of Huaishuo town in Guyang County, Baotou | 110°19′52" | 41°13′11" |
| S25 |  | Hougeertu village of Yinhao town in Guyang County, Baotou | 110°38′41.82" | 41°02′47.22" |
| S26 |  | Guanniuju gacha of Shiguai Disrict in Baotou | 110°15′9.03″ | 40°45′14.70″ |
| S27 |  | Zao Gully in Jiufeng Mountain in Tumd Right Banner, Baotou | 110°44′32.28" | 40°42′17.82" |
| S28 | Huhhot City | Deshengyingzi village of Hale town in Wuchuan County, Huhhot | 111°47′39" | 41°7′53" |
| S29 |  | Halaqin Gully in Daqingshan of Xin Cheng Disrict in Hohhot | 111°42′14.81″ | 41°0′22.89″ |
| S30 |  | Panjia Gully of Deshenggou town in Wuchuan County, Huhhot | 111°15′46″ | 41°1′25″ |
| S31 |  | Houyushu Gully of Deshenggou town in Wuchuan County, Huhhot | 111°13′35″ | 40°59′13″ |
| S32 |  | Xiaobei Gully of Daqingshan town in Wuchuan County, Huhhot | 111°30′30″ | 40°58′36″ |
| S33 |  | Xiaojing Gully of Xin Cheng Disrict in Hohhot | 111°50′13.82″ | 40°59′31″ |
| S34 |  | Xizuizi village of Chengguan town in Horinger County, Huhhot | 111°58′31.68″ | 40°25′54.89″ |
| S35 |  | Goumen village of Chengguan town in Horinger County, Huhhot | 111°57′7.56″ | 40°20′12.68″ |
| S36 |  | Xiheyao village of Xinyingzi town in tuoketuo County, Huhhot | 111°29′13.26″ | 40°11′44.85″ |
| S37 |  | Lidingyao village of Xinyingzi town in tuoketuo County, Huhhot | 111°30′51.40″ | 40°11′28.86″ |
| S38 |  | Houdajing village of Beibao town in Qingshuihe County, Huhhot | 111°45′5.82″ | 39°45′41.98″ |
| S39 |  | Houbeishiquan village of Beibao town in Qingshuihe County, Huhhot | 111°44′0.97″ | 39°47′45.68″ |
| S40 | Wulanchabu City | Balengshenla village of Hujitu town in Siziwang Banner, Wulanchabu | 111°56′26.7" | 41°22′56" |
| S41 |  | Bijiashan of Huofutan town in Siziwang Banner, Wulanchabu | 111°58′31" | 41°24′25" |
| S42 |  | Xifangzi village of Wulanhada sumu in Chahar Right Back Banner, Wulanchabu | 112°51′33.1" | 41°31′58.9" |
| S43 |  | Detailu village of Datan town in Chahar Middle Back Banner, Wulanchabu | 112°22′9.6" | 41°20′48.6" |
| S44 |  | Majiadi village of Xijingzi town in Shangdu County, Wulanchabu | 113°14′56.86″ | 41°43′17.00″ |
| S45 |  | Hongzhaojiulong Bay of Qixiaying town in Zhuozi County, Wulanchabu | 112°03′33.26" | 41°02′2.54" |
| S46 |  | Jinchengwa village of Shibatai town in Zhuozi County, Wulanchabu | 112°55′8.77″ | 40°56′17.04″ |
| S47 |  | Quanzigou village of Heitutai town in Feng Chin, Wulanchabu | 113°25′35.2" | 40°26′29.3" |
| S48 |  | Dahaowan village of Hongshaba town in Feng Chin, Wulanchabu | 113°14′5.6" | 40°40′7.8" |
| S49 |  | Zhanyangya village of Yuanshanzi town in Feng Chin, Wulanchabu | 113°32′41.3" | 40°26′43" |
| S50 |  | Mengjiawa village of Dakulian town in Xinghe County, Wulanchabu | 113°43′46.9″ | 41°12′27.2″ |
