# Supplementary material for: Influence of the Environment on the Distribution and Quality of Gentiana dahurica Fisch
Source: Front Plant Sci. 2021 Sep 27;12:706822. doi: 10.3389/fpls.2021.706822 (PMC8503573; doi:10.3389/fpls.2021.706822)
Supplement: Supplementary file 2 [file Table_2.docx]

Supplementary Material

# Supplementary Table S2

**Table S2.** The index component content of 50 *G. dahurica* samples $\boldsymbol{(}\bar{\boldsymbol{x}}\boldsymbol{\pm s, n=3)}$

| **NO.** | **loganic acid (ug)** | **6’-*O*-*β*-D-Glucosylgentiopicroside (ug)** | **swertiamarin (ug)** | **gentiopicroside (ug)** | **sweroside (ug)** | **total amount of loganic acid and gentiopicroside (ug)** | **total iridoids (ug)** |
| --- | --- | --- | --- | --- | --- | --- | --- |
| S1 | 0.8764±0.0053 | 3.3088±0.0205 | 0.3869±0.0023 | 3.7971±0.0309 | 0.0621±0.0028 | 4.6735 | 8.4313 |
| S2 | 0.8394±0.0079 | 0.945±0.0092 | 0.3483±0.0032 | 5.2706±0.0426 | 0.085±0.001 | 6.11 | 7.4883 |
| S3 | 0.3749±0.0039 | 0.9161±0.007 | 0.277±0.002 | 6.1633±0.0468 | 0.0323±0.0001 | 6.5382 | 7.7636 |
| S4 | 1.2669±0.0059 | 2.5816±0.0196 | 0.7554±0.0073 | 14.3366±0.0823 | 0.0886±0.0007 | 15.6035 | 19.0291 |
| S5 | 0.5552±0.0049 | 3.114±0.031 | 0.6819±0.0067 | 10.8658±0.1054 | 0.1168±0.0011 | 11.421 | 15.3337 |
| S6 | 0.6631±0.0048 | 8.0948±0.0593 | 0.8675±0.0073 | 10.2725±0.0824 | 0.0912±0.0018 | 10.9356 | 19.9891 |
| S7 | 0.3243±0.0121 | 1.2822±0.0472 | 0.2556±0.0084 | 4.1966±0.133 | 0.023±0.0014 | 4.5209 | 6.0817 |
| S8 | 0.6337±0.1217 | 1.1995±0.1039 | 0.4384±0.0543 | 7.5812±1.0198 | 0.0818±0.0056 | 8.2149 | 9.9346 |
| S9 | 0.7587±0.0043 | 1.4869±0.0072 | 0.5382±0.0066 | 7.2533±0.0615 | 0.1337±0.0023 | 8.012 | 10.1708 |
| S10 | 0.616±0.0211 | 0.5902±0.0293 | 0.616±0.0227 | 7.4907±0.2601 | 0.0623±0.0022 | 8.1067 | 9.3752 |
| S11 | 0.5±0.0005 | 3.1191±0.0053 | 0.7313±0.0012 | 14.5517±0.0371 | 0.0718±0.0001 | 15.0517 | 18.9739 |
| S12 | 1.0815±0.0051 | 6.5654±0.03 | 0.5124±0.0028 | 7.8787±0.0384 | 0.0906±0.001 | 8.9602 | 16.1286 |
| S13 | 0.9067±0.0028 | 2.3021±0.0147 | 0.474±0.0031 | 11.7372±0.0662 | 0.0735±0.0003 | 12.6439 | 15.4935 |
| S14 | 0.6059±0.0122 | 2.0521±0.0335 | 0.4323±0.0066 | 9.7103±0.1472 | 0.106±0.0019 | 10.3162 | 12.9066 |
| S15 | 0.5617±0.0009 | 2.8295±0.0116 | 0.4919±0.0024 | 11.2819±0.0372 | 0.083±0.0003 | 11.8436 | 15.248 |
| S16 | 1.1063±0.0211 | 2.3351±0.0376 | 0.4819±0.0076 | 11.7011±0.1793 | 0.0698±0.0011 | 12.8074 | 15.6942 |
| S17 | 0.7519±0.0026 | 1.7543±0.012 | 0.3699±0.0028 | 8.5399±0.0516 | 0.064±0.0005 | 9.2918 | 11.48 |
| S18 | 1.26±0.0173 | 2.1194±0.03 | 0.4225±0.0053 | 8.4921±0.097 | 0.1154±0.0016 | 9.7521 | 12.4094 |
| S19 | 1.0134±0.0075 | 1.8114±0.0075 | 0.5246±0.0032 | 11.8449±0.0526 | 0.1293±0.0018 | 12.8583 | 15.3236 |
| S20 | 1.32±0.0084 | 4.2248±0.0283 | 0.7855±0.0056 | 13.2982±0.0848 | 0.1211±0.0009 | 14.6182 | 19.7496 |
| S21 | 0.6812±0.0365 | 0.7168±0.0455 | 0.2692±0.0182 | 6.2652±0.4147 | 0.043±0.0027 | 6.9464 | 7.9754 |
| S22 | 0.3333±0.0108 | 3.0085±0.0851 | 0.4433±0.0121 | 7.8024±0.2079 | 0.049±0.0012 | 8.1357 | 11.6365 |
| S23 | 0.6586±0.0105 | 0.9298±0.0146 | 0.3012±0.006 | 6.8512±0.1188 | 0.0318±0.0009 | 7.5098 | 8.7726 |
| S24 | 0.2099±0.011 | 3.1394±0.1887 | 0.4403±0.0236 | 6.6674±0.3341 | 0.0574±0.003 | 6.8773 | 10.5144 |
| S25 | 0.7526±0.0118 | 3.0082±0.0218 | 0.5721±0.0045 | 10.1866±0.0567 | 0.0623±0.0004 | 10.9392 | 14.5818 |
| S26 | 0.4189±0.0064 | 1.7436±0.0174 | 0.5157±0.0049 | 9.6869±0.0795 | 0.0615±0.001 | 10.1058 | 12.4266 |
| S27 | 0.7687±0.0055 | 3.4935±0.0299 | 0.4714±0.0044 | 7.2591±0.0628 | 0.0545±0.0008 | 8.0278 | 12.0472 |
| S28 | 1.2251±0.0069 | 1.8439±0.0114 | 0.4387±0.0021 | 6.9947±0.0343 | 0.0437±0.0006 | 8.2198 | 10.5461 |
| S29 | 0.5494±0.0048 | 1.19±0.0043 | 0.3756±0.017 | 6.5423±0.0347 | 0.0725±0.0004 | 7.0917 | 8.7298 |
| S30 | 0.8221±0.0029 | 1.2934±0.0065 | 0.4165±0.0033 | 8.1259±0.051 | 0.0559±0.0005 | 8.948 | 10.7138 |
| S31 | 0.8011±0.0107 | 1.2244±0.0157 | 0.4579±0.0056 | 8.5097±0.0997 | 0.0613±0.0007 | 9.3108 | 11.0544 |
| S32 | 1.2644±0.0203 | 0.9144±0.01 | 0.4422±0.005 | 8.9468±0.0821 | 0.0625±0.0017 | 10.2112 | 11.6303 |
| S33 | 1.0206±0.0101 | 1.5151±0.011 | 0.5625±0.0047 | 9.9941±0.0846 | 0.0734±0.0008 | 11.0147 | 13.1657 |
| S34 | 1.3553±0.0676 | 0.9981±0.0478 | 0.4749±0.0225 | 9.799±0.44 | 0.0676±0.0035 | 11.1543 | 12.6949 |
| S35 | 0.5548±0.0065 | 2.3584±0.0076 | 0.5603±0.0041 | 10.4846±0.0754 | 0.0549±0.0002 | 11.0394 | 14.013 |
| S36 | 0.6473±0.007 | 1.9224±0.0169 | 0.4979±0.0043 | 9.2724±0.0941 | 0.0992±0.001 | 9.9197 | 12.4392 |
| S37 | 0.6476±0.0157 | 2.5076±0.0357 | 0.6275±0.0077 | 10.6315±0.1251 | 0.1044±0.0009 | 11.2791 | 14.5186 |
| S38 | 0.8915±0.0129 | 2.5763±0.0336 | 0.7451±0.0091 | 11.8694±0.2146 | 0.1039±0.001 | 12.7609 | 16.1862 |
| S39 | 1.0021±0.0076 | 2.1327±0.0245 | 0.6884±0.0072 | 11.5736±0.1295 | 0.1274±0.0017 | 12.5757 | 15.5242 |
| S40 | 0.7661±0.0071 | 1.9109±0.0234 | 0.4786±0.006 | 9.0771±0.1139 | 0.0473±0.0034 | 9.8432 | 12.28 |
| S41 | 0.9545±0.0339 | 1.463±0.0492 | 0.4035±0.0144 | 6.3399±0.1983 | 0.0475±0.0015 | 7.2944 | 9.2084 |
| S42 | 0.7567±0.0144 | 3.1674±0.0545 | 0.5185±0.0115 | 7.9798±0.1883 | 0.0544±0.0013 | 8.7365 | 12.4768 |
| S43 | 0.6629±0.0128 | 2.3085±0.0321 | 0.4466±0.0071 | 6.3195±0.0854 | 0.0511±0.0012 | 6.9824 | 9.7886 |
| S44 | 0.4322±0.0092 | 1.1742±0.0237 | 0.4198±0.0077 | 6.7098±0.1053 | 0.068±0.0012 | 7.142 | 8.804 |
| S45 | 1.3805±0.0025 | 3.7053±0.0051 | 0.5316±0.0016 | 8.8249±0.0232 | 0.0563±0.001 | 10.2054 | 14.4986 |
| S46 | 0.7388±0.0059 | 2.1856±0.0174 | 0.5717±0.0051 | 9.0527±0.0728 | 0.0859±0.0008 | 9.7915 | 12.6347 |
| S47 | 0.3654±0.0104 | 1.7774±0.058 | 0.5347±0.0195 | 6.6452±0.1871 | 0.0698±0.001 | 7.0106 | 9.3925 |
| S48 | 0.509±0.0073 | 0.9208±0.019 | 0.4069±0.0071 | 6.3665±0.0895 | 0.0776±0.0013 | 6.8755 | 8.2808 |
| S49 | 0.8058±0.0075 | 2.5304±0.018 | 0.5932±0.0047 | 9.3739±0.0801 | 0.0843±0.0009 | 10.1797 | 13.3876 |
| S50 | 0.2522±0.0104 | 1.0394±0.0399 | 0.2646±0.008 | 4.2075±0.1594 | 0.0353±0.0036 | 4.4597 | 5.799 |

Note: The 2020 edition of *Chinese Pharmacopoeia* stipulates that the total amount of loganic acid and gentiopicroside in this dried product shall contain not less than 2.5%.
