# Supplementary material for: Influence of the Environment on the Distribution and Quality of Gentiana dahurica Fisch
Source: Front Plant Sci. 2021 Sep 27;12:706822. doi: 10.3389/fpls.2021.706822 (PMC8503573; doi:10.3389/fpls.2021.706822)
Supplement: Supplementary file 3 [file Table_3.docx]

Supplementary Material

# Supplementary Table S3

**Table S3.** Soil type numbering table

| NO | Soil type |
| --- | --- |
| 1 | Urban |
| 2 | Inland water |
| 3 | Cambic Arenosols |
| 4 | Calcaric Cambisols |
| 5 | Humic Cambisols |
| 6 | Calcaric Fluvisols |
| 7 | Dystric Fluvisols |
| 8 | Umbric Gleysols |
| 9 | Calcic Kastanozems |
| 10 | Dystric Leptosols |
| 11 | Eutric Regosols |
| 12 | Haplic Solonetz |
| 13 | Dystric Cambisols |
| 14 | Eutric Cambisols |
| 15 | Ferric Podzols |
| 16 | Gleyic Cambisols |
| 17 | Luvic Chernozems |
| 18 | Haplic Chernozems |
| 19 | Calcic Chernozems |
| 20 | Rendzic Leptosols |
| 21 | Calcic Gleysols |
| 22 | Haplic Greyzems |
| 23 | Mollic Gleysols |
| 24 | Calcaric Phaeozems |
| 25 | Gleyic Phaeozems |
| 26 | Haplic Phaeozems |
| 27 | Haplic Kastanozems |
| 28 | Luvic Kastanozems |
| 29 | Terric Histosols |
| 30 | Gleyic Solonetz |
| 31 | Mollic Leptosols |
| 32 | Haplic Calcisols |
| 33 | Luvic Calcisols |
| 34 | Gleyic Solonchaks |
| 35 | Calcaric Regosols |
| 36 | Haplic Luvisols |
| 37 | Calcic Luvisols |
| 38 | Dystric Regosols |
| 39 | Haplic Arenosols |
| 40 | Stagnic Luvisols |
| 41 | Gleyic Luvisols |
| 42 | Haplic Gypsisols |
| 43 | Stagnic Phaeozems |
| 44 | Cumulic Anthrosols |
| 45 | Calcaric Arenosols |
| 46 | Aric Anthrosols |
| 47 | Gleyic Chernozems |
| 48 | Petric Calcisols |
| 49 | Luvic Gypsisols |
| 50 | Calcic Gypsisols |
| 51 | Calcic Vertisols |
| 52 | Eutric Leptosols |
| 53 | Salic Fluvisols |
| 54 | Mollic Solonchaks |
| 55 | Gypsic Solonchaks |
| 56 | Sodic Solonchaks |
| 57 | Calcic Solonchaks |
| 58 | Calcic Solonetz |
| 59 | Dunes & shifting sands |
| 60 | Fishpond |
| 61 | CHERNOZEMS |
| 62 | KASTANOZEMS |
