# Supplementary material for: Influence of the Environment on the Distribution and Quality of Gentiana dahurica Fisch
Source: Front Plant Sci. 2021 Sep 27;12:706822. doi: 10.3389/fpls.2021.706822 (PMC8503573; doi:10.3389/fpls.2021.706822)
Supplement: Supplementary file 4 [file Table_4.docx]

Supplementary Material

# Supplementary Table S4

**Table S4.** Vegetation type numbering table

| NO | Vegetation type |
| --- | --- |
| 1 | Typical grassland of temperate grasses |
| 2 | Temperate succulent saline dwarf semi-shrub desert |
| 3 | Temperate scrub dwarf grass and dwarf semi-shrub desert grassland |
| 4 | Temperate semi-shrub, short semi-shrub desert |
| 5 | None |
| 6 | Other |
| 7 | Temperate grasses and miscellaneous grasses saline meadows |
| 8 | Temperate shrub desert |
| 9 | Cold-temperate and temperate montane coniferous forests |
| 10 | Temperate deciduous scrub |
| 11 | Temperate grassland and mixed grass meadows |
| 12 | Temperate grasses, mosses and miscellaneous grasses swampy meadows |
| 13 | Temperate deciduous small-leaved sparse forest |
| 14 | Temperate dwarf semi-arboreal desert |
| 15 | Boreal and temperate swamps |
| 16 | Annual grain crops and hardy cash crops, deciduous fruit tree orchards |
| 17 | Temperate steppe shrub desert |
| 18 | Temperate coniferous forests |
| 19 | Annual short-fertility cold-tolerant crops (fruitless trees |
| 20 | Temperate deciduous broadleaf forest |
| 21 | Annual grain crops and cold-tolerant cash crops |
| 22 | Temperate grasses |
| 23 | Temperate grassy, mixed grass meadow grassland |
