# Supplementary figures and images for: Influence of the Environment on the Distribution and Quality of Gentiana dahurica Fisch
Source: Front Plant Sci. 2021 Sep 27;12:706822. doi: 10.3389/fpls.2021.706822 (PMC8503573; doi:10.3389/fpls.2021.706822)

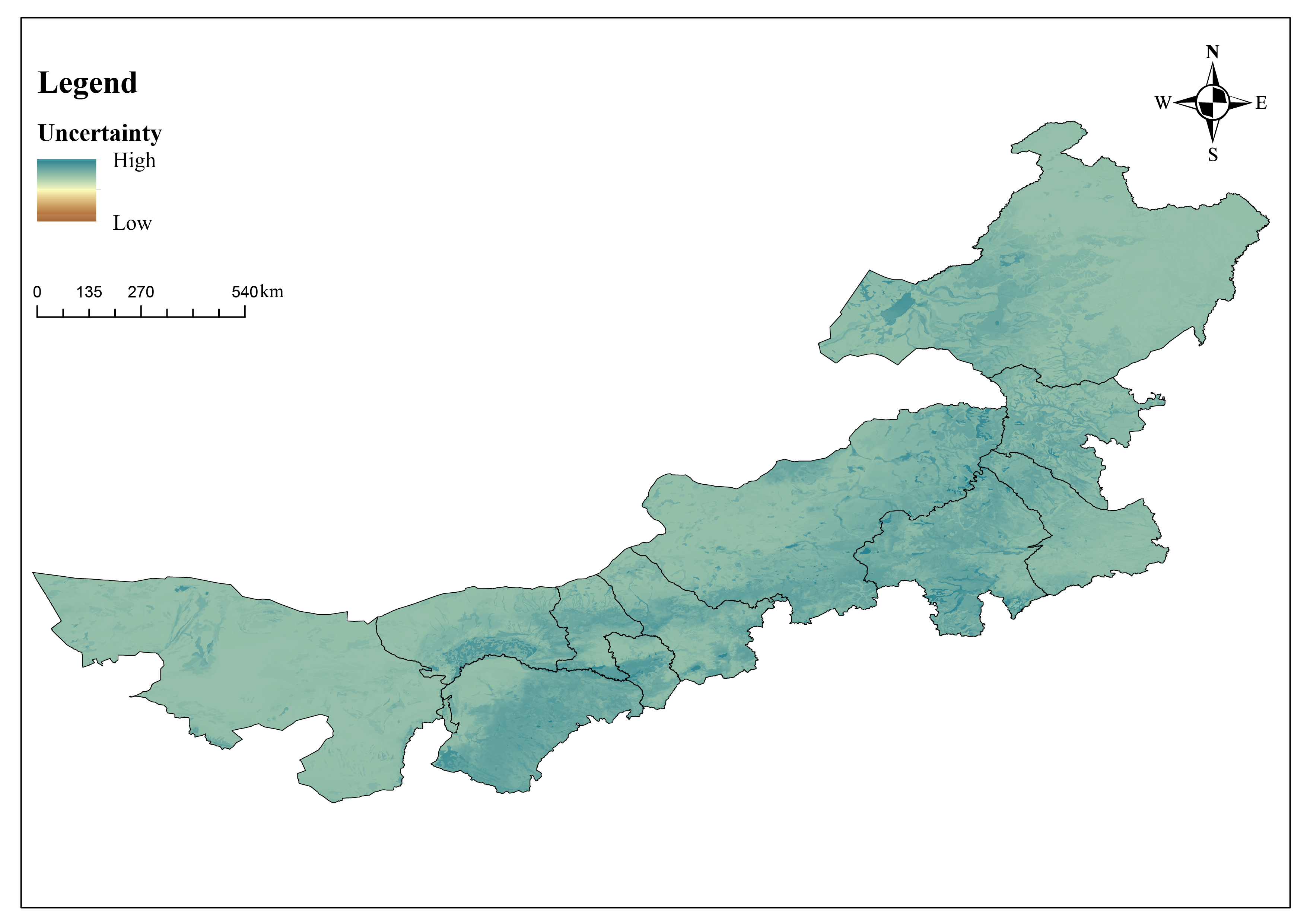

Supplement: Supplementary file 5 [file Image_1.JPEG]

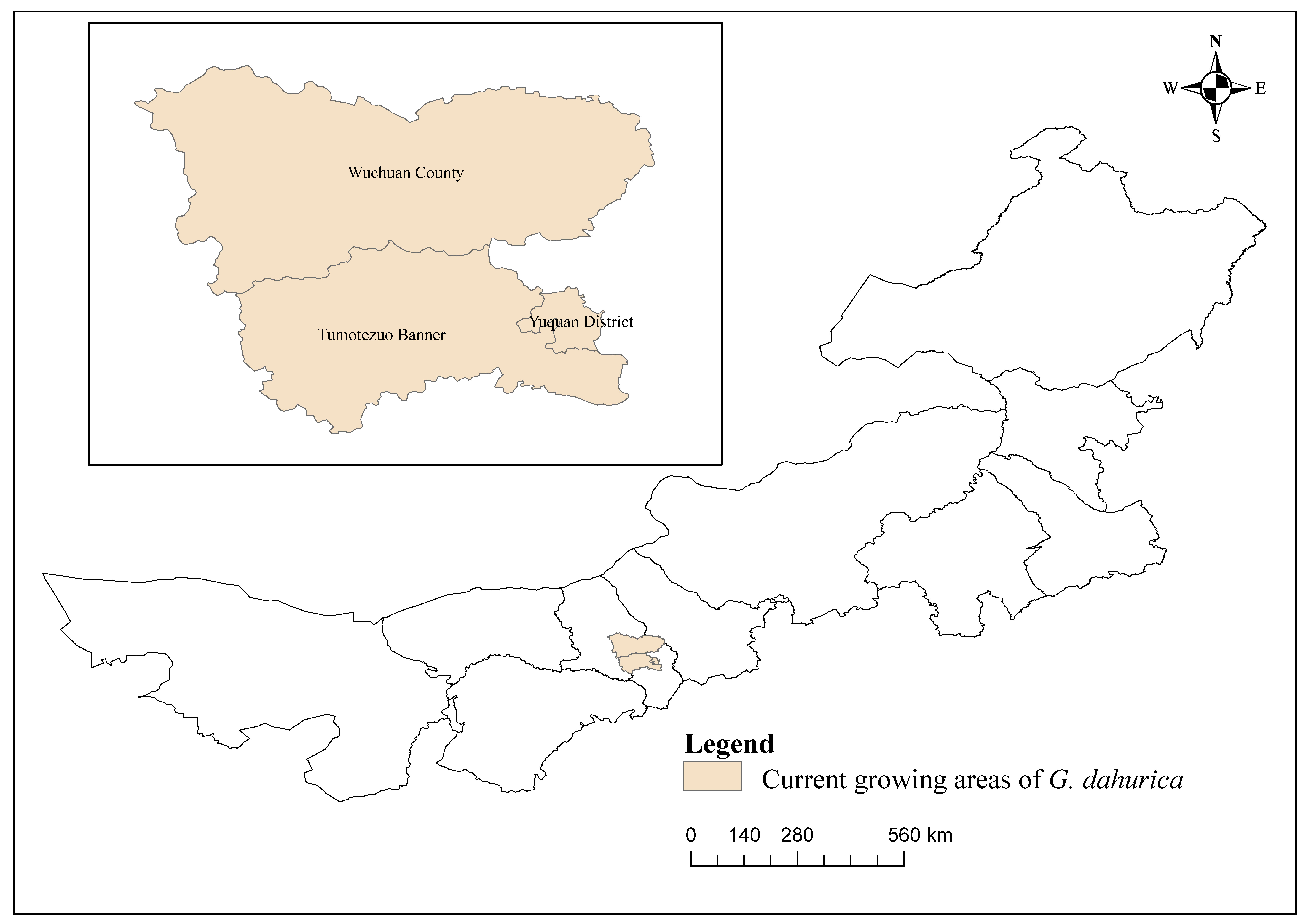

Supplement: Supplementary file 6 [file Image_2.JPEG]

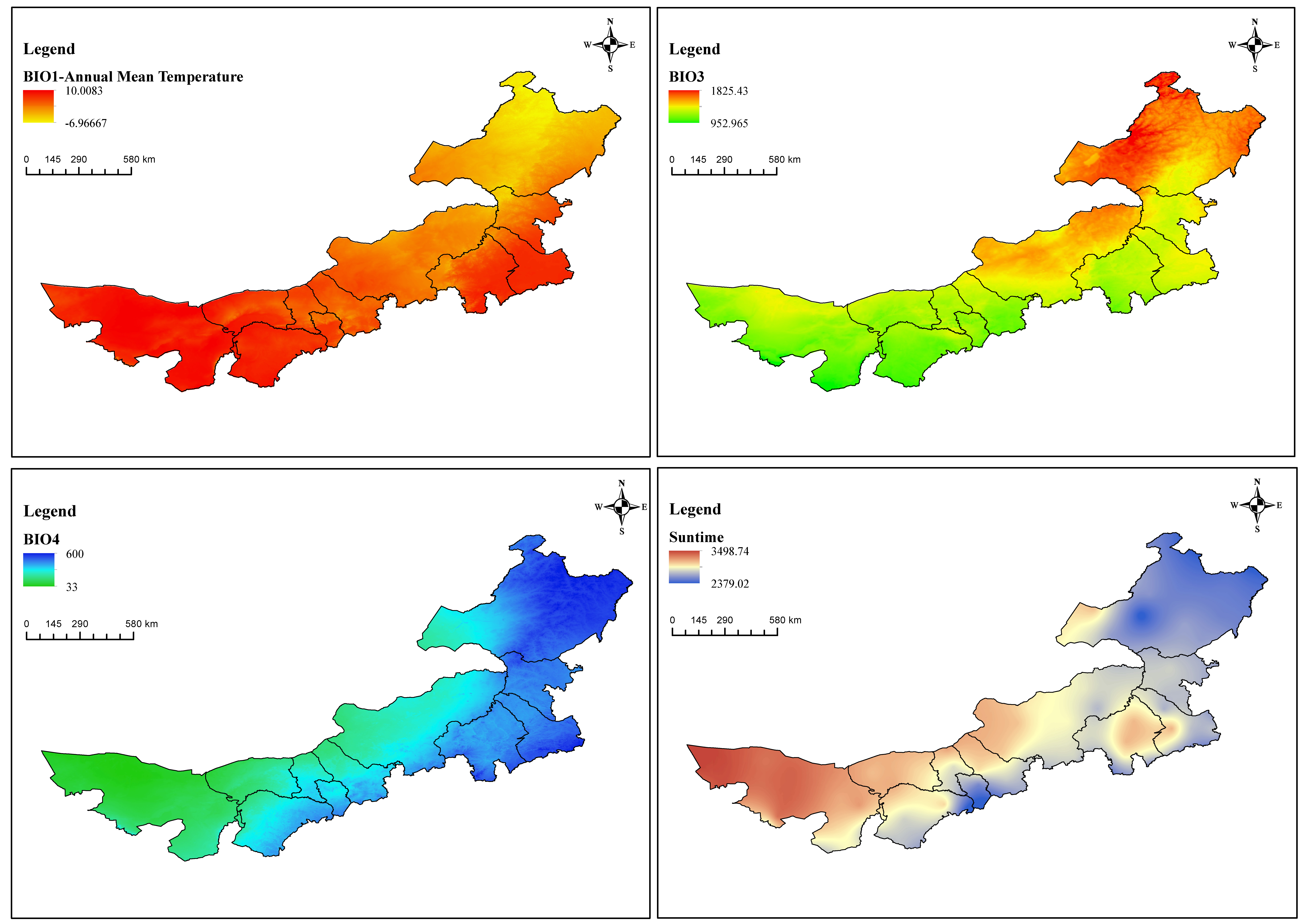

Supplement: Supplementary file 7 [file Image_3.JPEG]

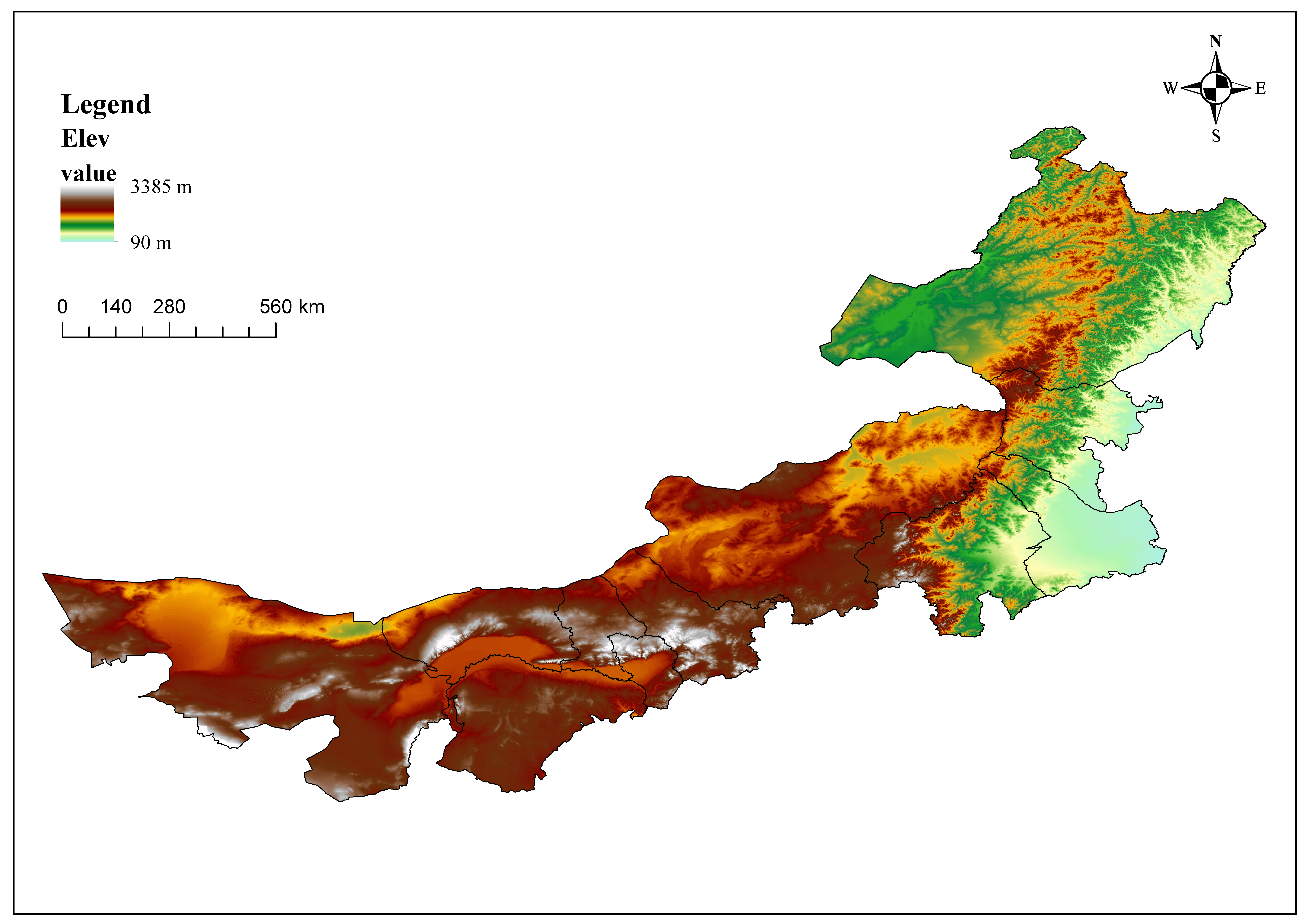

Supplement: Supplementary file 8 [file Image_4.JPEG]

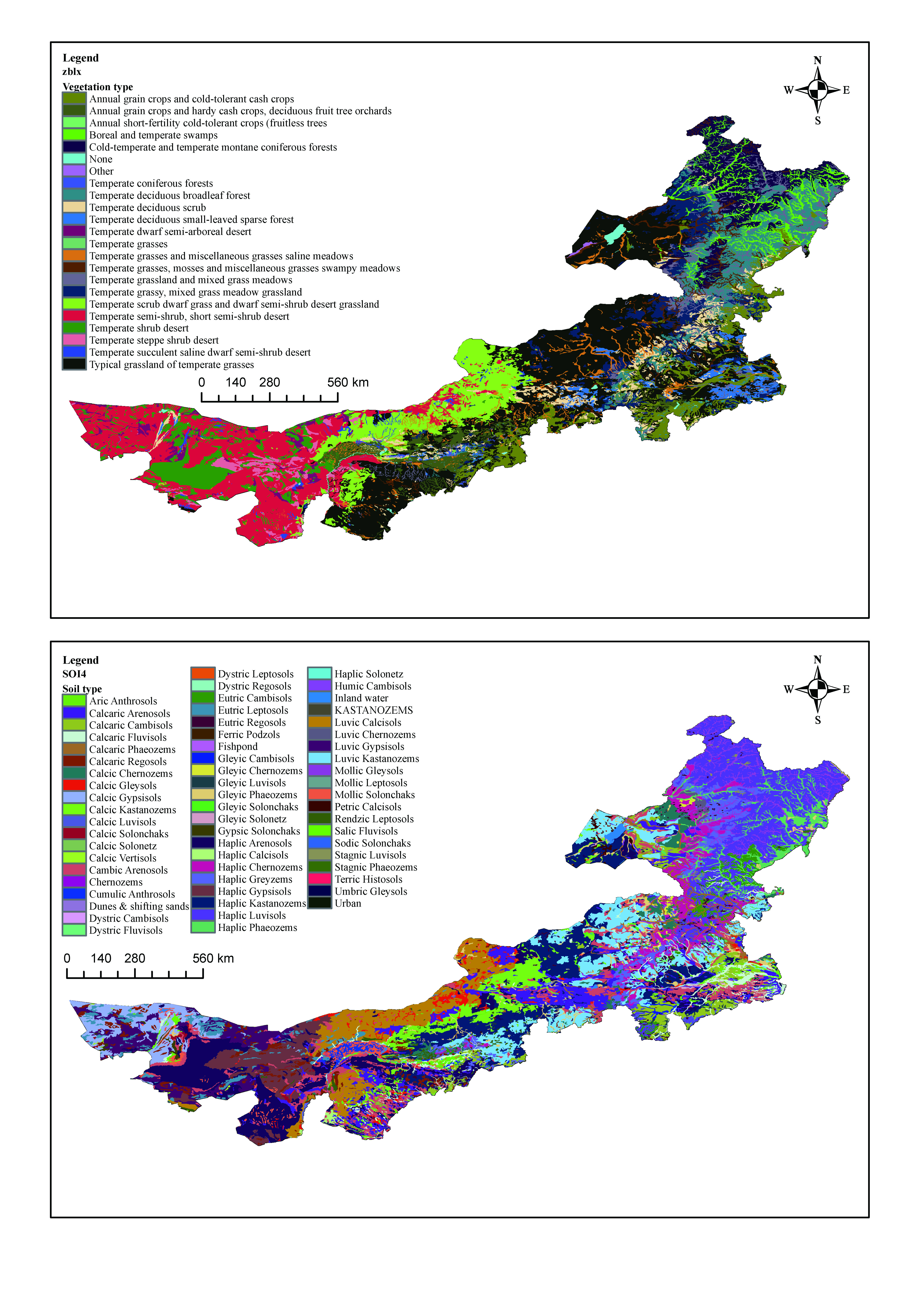

Supplement: Supplementary file 9 [file Image_5.JPEG]
